# Scientific review of protocols to enhance informativeness of global health clinical trials

**DOI:** 10.1186/s13063-025-08763-4

**Published:** 2025-03-12

**Authors:** B. Burford, T. Norman, S. Dolley

**Affiliations:** 1 National Coalition of Independent Scholars, Battleboro, USA; 2 The Bill & Melinda Gates Foundation, Seattle, USA; 3 Open Global Health, Arlington, USA

**Keywords:** Informativeness, Global health, Clinical trials, Protocol, Content analysis, Text classification, Frequency analysis, Scientific review

## Abstract

**Background:**

Trial informativeness describes the likelihood of a clinical trial to have a meaningful impact on clinical practice, research, or policy decisions. A dedicated scientific review process for protocols at the post-funding stage is not common, yet is an opportunity to enhance trial informativeness. The Bill and Melinda Gates Foundation (BMGF), one of the largest funders of clinical trials, created a group called Design, Analyze, Communicate (DAC). DAC’s first completion of an expert scientific review of a grantee’s trial protocol was in 2020. We categorized and quantified areas of scientific review feedback provided for 52 clinical trial protocols submitted to DAC over a 3-year period. Most trials planned to study treatment interventions and included at least one trial site in a low- and middle-income country. Feedback themes offer insight into areas of trial design weakness.

**Methods:**

We conducted a retrospective analysis of protocol review feedback provided by DAC to grantees. Reviews were completed by BMGF between 2020 and 2022. A qualitative content analysis was conducted by developing a codebook of clinical trial methodology topics and subtopics and systematically coding free-text review feedback. Manual text classification of individual feedback statements enabled quantification and frequency analysis of review feedback.

**Results:**

A total of 1537 individual recommendations were made across all 52 protocols. The median number of recommendations per protocol was 28 (range: 13 to 52), covering a wide range of issues related to clinical trial design, implementation, analysis, and impact. Nearly half of all recommendations (47%) were characterized by the review team as high priority. The areas with the highest frequency of recommendations were statistics and data analysis, trial procedures, and intervention/dose.

**Conclusions:**

This study provides a taxonomy of scientific review feedback topic areas that can be used to categorize clinical trial design topics. The high number of recommendations per protocol review across several distinct topic areas highlights the need for a scientific review to enhance trial informativeness. This review must take place prior to trial initiation and review teams should include statistical and trial design expertise with additional expertise tailored to the trial/intervention type and phase.

**Supplementary Information:**

The online version contains supplementary material available at 10.1186/s13063-025-08763-4.

## Background

Informativeness, in the context of clinical research, is a term used to characterize how likely it is that the results of a trial will have a worthwhile impact on future decisions. An informative trial “provides robust clinical insight and a solid on-ramp to either the next phase of development, a policy change, a new standard of care, or the decision not to progress further [1].” An uninformative trial is “one that provides results that are not of meaningful use for a patient, clinician, researcher, or policy maker [2].” Clinical trials that end with the quality of informativeness must answer a meaningful question, have met their recruitment goal, answered their primary research question with sufficient power, and be designed to be accepted in the systematic reviews that are used by policymakers such as the World Health Organization (WHO) [2, 3]. When a funder invests in a clinical trial, and that trial never ends, or ends uninformatively, losses may include many years of delay in bringing treatments to patients; the loss of that trial’s participants’ time and faith in science; the question of whether the funder met their fiduciary duty to investors or donors; and the opportunity cost of losing out on conducting a more informative trial in its place.

Lack of informativeness may be a particular risk in non-industry-sponsored studies. A recent analysis suggested that 74% of trials overall did not meet four essential criteria for informativeness. They found that trials sponsored by industry were much more likely to fulfill informativeness criteria than trials with non-industry sponsorship (50% vs. 6%) [3]. It is imperative that sufficient resources are invested prior to the commencement of a clinical trial to avoid future waste, with a focus on optimizing trial design and methods. A sample of 200 trials included in Cochrane review meta-analyses, that had at least one domain assessed to be at high risk of bias, were found to include 25 types of methodological problems [4]. The authors deemed that methodological adjustments were possible in 96% of trials, and they estimated “avoidable waste due to inadequate methods” to be at 42%. Furthermore, there are likely trials with more significant methodological problems that did not meet the criteria for the Cochrane reviews included in that analysis.

Review processes prior to the start of a trial provide an opportunity to enhance informativeness. Post-funding scientific reviews of individual trials differ from the more typical pre-funding peer review process [5]. Peer reviews of prospective research are well-established, as well as ethics and regulatory reviews. A pre-funding review is typically focused on differentiating from a large pool of grant applications. Grant applications considered in pre-funding peer review may relate to a single trial, or they may encompass several potential trials and are not designed to provide the same level of detail as a trial protocol. Peer review of grant applications is focused on whether or not the research should be funded and includes consideration of a wide range of factors related to this investment decision [6–9]. As such, peer review is not likely to provide a comprehensive assessment and feedback about the ways that the study design and conduct could change to maximize informativeness. Despite this apparent gap that could be addressed by a scientific review post-funding, this type of review is not commonplace. Post-funding reviews of global health clinical trials—studies focused on generating robust evidence on medical interventions for all, but especially for those in low-resource settings—usually only include an ethics review and/or regulatory review. These reviews often do not have the bandwidth, mission, or capacity to focus on methodological issues, scientific integrity or informativeness [10]. There is acknowledgement that some assessment of scientific merit is generally considered necessary in an ethics review; there remains debate about whether scientific design reviews ought to occur completely within the ethics review [11]. Some organizations conduct an independent scientific review of clinical trial protocols internally. For example, the Intramural Research Program of the United States (US) NIH (National Institutes of Health) has a policy of conducting a scientific review of all protocols prior to review by the NIH Institutional Review Board (IRB) [12]. This scientific review focuses on many aspects including scientific merit, objectives, design, study population, outcomes and endpoint definition, statistical analysis, sample size, and data monitoring. A distinct scientific review stage for protocols is also common among cancer trials sponsored by US academic cancer centers [13, 14].

The Bill and Melinda Gates Foundation (BMGF), an organization whose mission is to “create a world where every person has the opportunity to live a healthy, productive life” [15], is one of the largest non-industry funders of clinical trials in the world. To maximize the positive contribution of BMGF-funded trials in line with their mission, BMGF created a group called Design, Analyze, Communicate (DAC) in 2019. DAC, a domain within the Global Health division, is designed to perform scientific reviews of protocols and give BMGF grantees insights, tools, and resources to improve clinical studies that BMGF has funded. DAC’s first completed scientific design review was in 2020. The proportion of clinical trials conducted in low- and middle-income countries (LMICs) in traditional global health disease areas (communicable, maternal, neonatal, and nutrition) is typically much smaller compared with the proportion conducted in high-resource settings in disease areas of greater concern in those settings (e.g., non-communicable diseases), meaning it is arguably even more critical that the former are given every opportunity to have a meaningful impact. The benefit of DAC is to de-risk, optimize, and accelerate the pipeline of drugs, vaccines, and other interventions headed to low- and middle-income populations, by decreasing research fails. DAC has assembled a team of clinical research experts in many focus areas to assess trial protocols. A large fraction of these experts are external to BMGF, which not only reduces the likelihood of a conflict of interest, but enables BMGF to leverage a diversity of expertise tailored to the needs of individual review activities.

A DAC protocol review (PR) typically happens post-funding and requires a draft trial protocol. In addition, grantees provide supporting documents and complete an assessment questionnaire [16]. The reviewers, according to their area of expertise, provide recommendations on ways a grantee might improve the protocol from a scientific and feasibility perspective. DAC defines trial informativeness as shown in Table 1. Reviewers are also pointed to a selection of trial “best practice” validated research methods assembled by DAC that may increase the likelihood of achieving “trial informativeness” (see Table 2). Reviewer recommendations are provided in the format of “items to consider.” While not required to adopt the expert recommendations, grantees can use the technical advice as they choose for protocol finalization.

**Table 1:** DAC definition of trial informativeness

An informative trial is designed to have the best chance to complete on time, answer its research questions definitively, and effect policy change or a go/no-go decision, by means of:
1. Locating trial sites based on epidemiology and impact rather than convenience,
2. Completing a statistical analysis plan concurrently with the trial protocol,
3. Using accepted endpoints and conservative effect and prevalence/incidence estimates, and
4. Utilizing contemporary techniques, such as statistical simulation, innovative trial designs, and software to monitor recruitment

**Table 2:** DAC best practices for trial informativeness

1. Prioritize disease burden and epidemiology as criteria for study site selection
2. Use accepted and validated endpoints whenever possible
3. Proactively map study outcome to immediate or ultimate policy impact
4. Rigorously justify effect estimates and prevalence assumptions
5. Simulate trial to ensure right sample size and optimal design
6. When feasible and relevant, apply adaptive, pragmatic, platform, or other innovative clinical trial designs
7. Analyze real-world evidence to optimize study investments, objectives, and feasibility
8. Prior to study initiation, complete a prospective, fixed statistical analysis plan
9. Design interim analyses with decision rules for stopping for success or futility early enough to reduce the number of participants subjected to ineffective interventions
10. When appropriate, use model-informed drug development, such as PK/PD modeling
11. Adhere to appropriate standards of randomization, blinding, allocation concealment, and reducing bias
12. Use staff with experience in the therapeutic area being studied
13. Implement a real-time data analysis capability, toward improved monitoring of recruitment targets, data quality, and other metrics
14. Engage local regulators, ethics committees, and policymakers before, during, and after the study, for input on design, obtaining relevant approvals, and action at study’s end
15. Implement a communication plan and informed consent that involves participants, families, communities, and health systems
16. Publish protocol, analysis plan, and study results, including raw study data and code, in an open access resource, regardless of study outcome

Review panels are usually comprised of a group of external subject matter experts (SMEs). On every review, there is typically a clinical pharmacology and biostatistics SME, with additional SMEs depending on the specialized areas appropriate for each protocol. These specialized areas have included bioinformatics, biologics, clinical operations in Africa, epidemiology, genetics, global health policy, health economics, implementation research, infectious disease, nutrition, pharmacology, physiologically-based pharmacokinetics, regulatory affairs, and vaccine science.

Very few studies have published information characterizing protocol review feedback provided to protocol authors, particularly from a methodological perspective. One study analyzing NCI-mandated scientific review of clinical trial protocols at a cancer center found that the process resulted in changes to the study design and intervention in 40% of investigator-initiated studies [17]. In the current study, we categorized and quantified the areas of feedback provided in response to the completion and availability of full documentation of all reviews (*n* = 52) in a 3-year period, 2020–2022, across a range of disease areas and interventions, using a process of codebook development for content analysis by manual text classification. Knowledge of feedback themes can offer insight into the types of issues that should be considered by grantees and scientific review panels. Understanding potential areas of trial design weakness may also be helpful when considering the types of support best offered in the early stages of trial design and protocol development for global health clinical trials to increase informativeness and ensure wise funding decisions are made.

## Methods

**Fig. 1:**
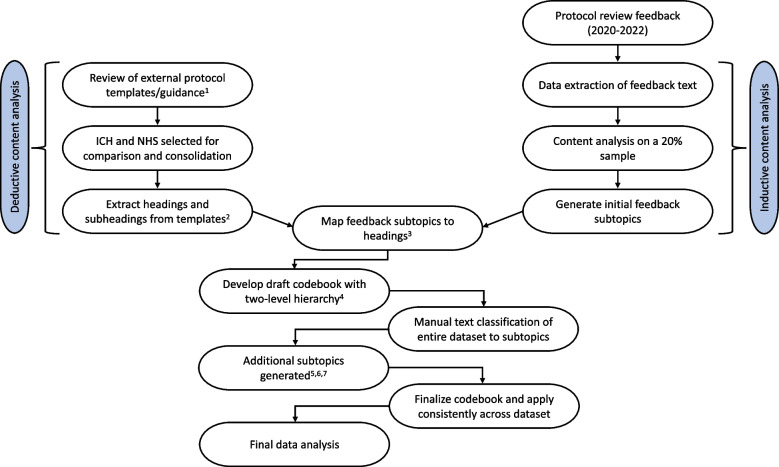
Methodological steps in developing a codebook for manual text classification of feedback statements. ^1^Protocol development guidance and templates from major research authorities (International Council for Harmonisation of Technical Requirements for Pharmaceuticals for Human Use (ICH), WHO, Clinicaltrials.gov, United Kingdom (UK) National Health Service (NHS) Health Research Authority) were reviewed. ^2^Headings and subheadings from both ICH and NHS clinical trial protocol templates were extracted to identify the topics included and their organization within both documents. ^3^Subtopics generated from the initial analysis of source data were mapped to ICH and NHS protocol template headings. ^4^Feedback subtopics were thematically categorized into a fewer number of broader topics at the high level. ICH and NHS templates informed the organization of this hierarchy. ^5^Topics that had only a single subtopic and accounted for 2% or more of recommendations were subdivided into more specific subtopics (informed by subheadings within ICH protocol guidance) to increase granularity. ^6^One topic (“Intervention/Dose”) initially had only three subtopics, but as it accounted for 10% of recommendations overall, “Intervention/Dose” was subdivided into additional subtopics to provide more detail. ^7^A small number of additional subtopics were defined using a consensus approach to incorporate any recommendations where it was established that they did not fit within existing subtopic definitions

We conducted a retrospective analysis of protocol review feedback provided by DAC expert review teams to grantees of 52 trial protocols within the 3-year period 2020–2022. This number represented all the protocols whose documentation and reviews were completed in the period; one review, the 53rd, did not have finalized documentation available, and as such was not included. A qualitative content analysis was conducted by developing a codebook of clinical trial methodology topics and subtopics and systematically coding free-text review feedback. Manual text classification of individual feedback statements enabled quantification and frequency analysis of review feedback. This process is shown in Fig. 1 and described in detail below.

### Source data

The protocols for this analysis were reviewed with completion dates between 2020 and 2022. Post-funding scientific reviews were conducted by an expert panel comprised of a nominal presence of BMGF staff, as well as SMEs external to BMGF. Information documented as part of the DAC project management process, including review timelines, composition of review teams, study type, and trial phase, was collected to describe the characteristics of the protocol reviews in this analysis. The median number of SMEs on each review panel was 5 (range: 3 to 8). Beyond clinical, regulatory, biostatistics, and pharmacology expertise, there were particular areas of focus for SMEs as relevant to each trial protocol including PK/PD, quantitative sciences, epidemiology, vaccines, policy, Africa research operations, implementation, nutrition, health economics and outcomes research, and digital health. This is not an exhaustive list as there was considerable variation in the trials and many experts had additional areas of expertise.

Protocol review feedback from DAC was provided in a document with a standardized format and structure called an “Opportunities List” (OL). Feedback is provided as “Comments from DAC” with an “Action Recommended from DAC.” The structured format of the OL enhances the clarity of review feedback. The separate “Comments” and “Action recommended” sections guide reviewers to ensure that any potential problems identified with a protocol are accompanied by recommended solutions. Each area of feedback is prioritized by criticality into high, medium, or low priority. High priority means that DAC believes the trial is at risk of ending uninformatively unless the issue is addressed before the study starts. Medium priority means that DAC is flagging an issue that may increase the risk of an uninformative study outcome. Low priority is reserved for issues that do not represent a significant risk to informativeness, but if addressed could improve the overall clarity or rigor of the protocol. The raw data for this analysis consisted of all text included in the table component from 52 OLs with dates of delivery to grantees from 2020-2022. Granular data is not available as OLs are confidential.

### Data preparation

Each OL was reviewed by one of two authors (BB, SD) who extracted all text included in “Comments from DAC” as well as “Action Recommended from DAC” and parsed individual statements from those sections into recommendation statements that would be coded to subtopics. Assignment of OLs to one of two authors was carried out using a randomly generated numbering system. Extracted text was converted into specific, independent, action-oriented recommendation statements. This meant that the original wording was altered where necessary, while retaining the meaning of the source text. This was carried out to emphasize the action, and to make explicit the interpretation of the text used to code to a subtopic for this analysis. Sometimes recommended actions in the source data were grouped together to describe more than one related recommendation in one sentence, paragraph, or in a bulleted list. To increase transparency and reproducibility, and to accurately measure the number of recommendations, all review feedback text was read line-by-line and parsed into individual recommendation statements to enable clearly delineated topic selection, and to ensure that each individual recommendation was counted. Generally, recommended actions were split into more than one recommendation statement where it was conceivable that the grantee could carry out one recommendation and not the other.

### Codebook development

A codebook approach was taken to guide content analysis of the recommendations, so that each expert recommendation statement could be coded to an individual topic or theme that best suited the action that was recommended to be taken by the grantee. A two-level hierarchical approach was used so that the more granular level called “subtopics” were thematically categorized into a fewer number of broader “topics” at the high level. Initial subtopics were generated using a combination of manual text analysis and knowledge of standard terminology in clinical trial design and protocol development. An inductive content analysis was conducted first on a sample of OL documents (20%) to identify themes and topics that were discussed in the protocol review feedback, acknowledging that this process was influenced by the researchers own experience with known topic areas in research methods and clinical trial protocol development. Following this analysis, to inform the “topic” level of the taxonomy using a deductive content analysis approach, protocol development guidance, and templates from major research authorities (International Council for Harmonisation of Technical Requirements for Pharmaceuticals for Human Use (ICH), WHO, Clinicaltrials.gov, United Kingdom (UK) National Health Service (NHS) Health Research Authority) [18–22], were reviewed. Of these, the ICH protocol guidance was the most recently released (2022) [18]. The most similar to the new ICH protocol development template was the NHS Health Research Authority Protocol Development Template [22], so both of these templates were used to generate a set of high-level topics and inform the organization and definition of subtopics that were identified from the source data. Our combining of aspects of both inductive and deductive content analysis approaches was carried out to ensure that the resulting codebook would be adequately comprehensive to suit the source data while also aligning with well-established standards in clinical trial terminology and protocol development. This process is described in detail in Additional File 1.

### Manual text classification

For this content analysis, a manual text classification process was used to assign recommendation statements to subtopics in alignment with the definitions provided in the codebook. For example, the “Interim analysis” subtopic was defined as “Recommendations related to conducting an interim analysis, including for the purpose of sample size re-assessment, or stopping the trial for futility, success or safety reasons.” Each recommendation statement was categorized by one of two coders (BB, SD) to the most appropriate subtopic for the action recommended to the grantee who submitted the original trial protocol for review. For each recommendation, the coder entered a subtopic along with a level of certainty (high, medium, or low) for their subtopic choice. The level of certainty represented how confident the coder was that their assignation was correct. For a certainty rating of medium or low, a potential second subtopic choice was entered as a best candidate for replacement if the first subtopic choice was deemed inappropriate. A low certainty rating meant that the coder was only moderately sure of their subtopic assignment. All subtopics given a low certainty rating were reviewed by a second coder. In these instances, the second coder could choose to use either the subtopic choice proposed by the first coder or propose a new subtopic choice. If a new subtopic choice was proposed, this conflict was resolved using a consensus approach with the decision and reason noted. Coders also identified recommendations that were significantly statistical in nature, and an external biostatistician was consulted to assess these recommendations and make a final decision about subtopic allocation. See Additional File 1 for further detail about how the codebook was refined following initial manual text classification. A sample of PRs (50%) were coded independently by two coders and compared to estimate the inter-rater reliability of our codebook approach. The final codebook of 13 topics and 58 subtopics is reproduced in Additional File 2.

Overall, a total of 1610 recommendations were classified into subtopics. Seventy-three (73) of these were related to identifying grammar, spelling, formatting, or other inconsistencies in the document that were not independent recommendations for improving the trial or enhancing its impact on clinical practice and/or policy. Removing these left a total of 1537 recommendations classified into subtopics. Finally, the topic level hierarchy was mapped to the subtopics, and frequency analyses of topics and subtopics were conducted.

## Results

Fifty-two trial protocols that were reviewed by DAC between 2020 and 2022 were analyzed. Table 3 shows the protocol characteristics. The number of reviewers per review ranged from 3 to 8. The frequency of the mix indicates it was more typical to perform a review 30–60 days in duration with 5–6 reviewers. With the pressure for speed in drug development timelines, it seems likely that more interest would accrue to the capacity for faster review execution. This data shows more reviews taking less than 30 days than more than 60 days.

**Table 3:** Characteristics of clinical trial protocols undergoing DAC scientific review (2020–2022)

Characteristic	Number (%)
Study type	
Interventional	42 (81)
Non-interventional	10 (19)
Interventional purpose (*n* = 42)	
Prevention	16 (38)
Treatment	22 (52)
Health services research	2 (5)
Device feasibility	1 (2)
Other	1 (2)
Phase	
Phase 1	8 (15)
Phase 2	10 (19)
Phase 3	10 (19)
Phase 4	11 (21)
No phase	13 (25)
Adaptive trial design	
Yes	8 (15)
No	44 (85)
Trial has one or more trial sites in LMIC^a^	
Yes	47 (90)
No	5 (10)
Year	
2020	11 (21)
2021	14 (27)
2022	27 (52)
Number of SME reviewers	
3–4	18 (35)
5–6	26 (50)
7–8	8 (15)
Length of review period (business days)	
< 30 days	19 (37)
30–60 days	23 (44)
>60 days	10 (19)

^a^LMIC (low- and middle-income countries) defined according to World Bank country classifications by income level (2022–2023) [23]

After the removal of recommendations related specifically to inconsistencies or errors in documentation, a total of 1537 individual recommendations were made by expert reviewers across all 52 protocols submitted for review. The median number of recommendations per protocol was 28 (range: 13 to 52), with concerns raised covering a wide range of topics related to clinical trial design, implementation, analysis, and impact. The median number of distinct codebook topics raised per PR was 8 (range: 5 to 13).

### Protocol review recommendations

Each recommendation was coded once to a specific subtopic, out of a possible 58 subtopic choices. This was performed according to consistently applied definitions as outlined in the Methods. The inter-rater reliability was assessed using a 50% sample of the PRs (*n* = 802 recommendations). The percent agreement at the subtopic level was 78.7% (Cohen’s kappa 0.78 (95% CI: 0.75 to 0.81), which is generally considered to be substantial [24] or excellent [25] agreement. As expected, at the topic level, the percent agreement was higher at 87.0% (Cohen’s kappa 0.85 (95% CI, 0.82 to 0.87), which is generally considered to be excellent to near-perfect agreement.

**Fig. 2:**
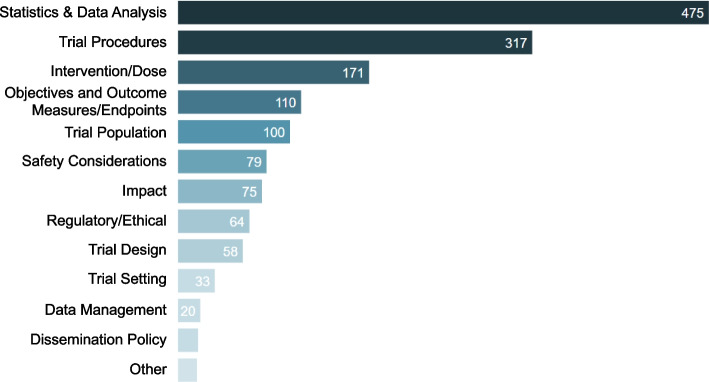
Protocol recommendation count by topic

Complete counts for the number of recommendations falling under each subtopic are shown in Additional File 3. Table 4 shows recommendation counts by subtopic stratified by priority and in descending order by the number of high priority recommendations. By frequency, the top 50% of the 1537 recommendations were assigned to a total of 13 different subtopics. Subtopics were categorized into higher level topics. Examining recommendation counts by topic shows that 52% of the recommendations fell into the top two topics: statistics and data analysis and trial procedures. Figure 2 shows the breakdown of recommendation count by topic. Only one topic, statistics and data analysis was raised in the feedback for every PR, closely followed by trial procedures, which was raised in 98% of PRs (Table 5).

**Table 4:** Recommendation counts by subtopic stratified by priority, shown in descending order by the number of high priority recommendations

Topic	Subtopic	High	Medium	Low	Total
Objectives and outcome measures/endpoints	Outcome measures and endpoints	48	20	14	82
Trial procedures	Data collection	42	32	26	100
Statistics and data analysis	Endpoint analysis	39	27	13	79
Statistics and data analysis	Sample size and power	32	19	7	58
Trial design	Design change	30	7	1	38
Statistics and data analysis	Adjusted analysis	28	36	4	68
Trial population	Inclusion and exclusion criteria	26	20	10	56
Trial procedures	Randomization	25	16	10	51
Statistics and data analysis	Analysis-other	24	23	15	62
Impact	Policy planning	24	5	1	30
Objectives and outcome measures/endpoints	Objectives	23	5		28
Statistics and data analysis	Statistical simulations	22	13		35
Intervention/dose	Intervention	21	26	1	48
Trial procedures	Implementation and feasibility	19	21	11	51
Impact	Stakeholder engagement	18	6	6	30
Trial population	Population selection	17	18	9	44
Intervention/dose	Dose selection	17	4	1	22
Intervention/dose	PK/PD	16	17	5	38
Statistics and data analysis	Interim analysis	16	8	1	25
Safety considerations	AE and SAE monitoring	15	15	6	36
Safety considerations	Safety-other	13	8	3	24
Statistics and data analysis	Estimates of effect	13	7	1	21
Trial setting	Site selection	13	6	1	20
Statistics and data analysis	Statistics-other	12	7	7	26
Trial design	Design description and rationale	12		1	13
Regulatory/ethical	Stopping rules	11	6	3	20
Regulatory/ethical	Trial monitoring	10	8	2	20
Trial procedures	Sample collection	9	18	10	37
Intervention/dose	Dose schedule and administration	9	5	2	16
Impact	Product development plan	9	3	3	15
Trial procedures	Enrollment	8	12	4	24
Statistics and data analysis	Estimates of prevalence	8	6	4	18
Intervention/dose	Controls, comparators	8	4	1	13
Trial procedures	Screening	7	9	1	17
Trial setting	Site criteria	7	6		13
Statistics and data analysis	SAP	6	9	8	23
Safety considerations	Safety assessments	6	12	1	19
Other	Other bias	6	3		9
Regulatory/ethical	Consent	5	7	7	19
Intervention/dose	Prep, handling, storage	5	3		8
Trial design	Design timepoints	5	2		7
Statistics and data analysis	Missing data	4	29	1	34
Data management	Data management	4	8	8	20
Intervention/dose	Intervention compliance	4	5	2	11
Other	Other	3	4	1	8
Trial procedures	Baseline assessments	3	4		7
Trial procedures	Retention	2	6	1	9
Intervention/dose	Concomitant therapies	2	2	2	6
Intervention/dose	Toxicity	2	1	1	4
Intervention/dose	Intervention-other	2	2		4
Statistics and data analysis	Subgroup analysis	1	15	10	26
Trial procedures	Community engagement	1	11	3	15
Regulatory/ethical	Ethical considerations	1	4		5
Trial procedures	Withdrawal criteria	1		2	3
Trial procedures	Long-term follow-up	1	1	1	3
Dissemination policy	Open access		9	5	14
Dissemination policy	Dissemination		1	3	4
Intervention/dose	Dose-other		1		1
	**Total**	**715**	**582**	**240**	**1537**

**Table 5:** The number and proportion of protocol reviews (PRs) in the dataset that included recommendations for each topic

Topic	Number (%) of PRs
Statistics and data analysis	52 (100)
Trial procedures	51 (98)
Trial population	44 (85)
Intervention/dose	43 (83)
Objectives and outcome measures/endpoints	43 (83)
Safety considerations	39 (75)
Impact	36 (69)
Regulatory/ethical	36 (69)
Trial design	34 (65)
Trial setting	22 (42)
Other	16 (31)
Dissemination policy	15 (29)
Data management	10 (19)

### Recommendations by priority

**Fig. 3:**
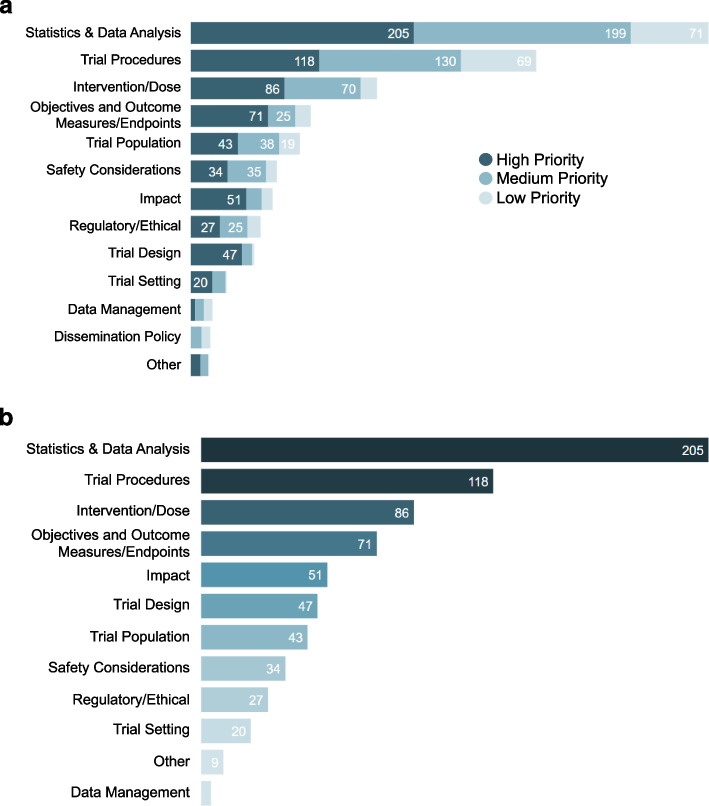
**a** Protocol recommendation count by priority within topic (*n* = 1537). **b** Protocol recommendation count by topic, high priority (*n* = 715)

All DAC protocol review recommendations, when provided to grantees and internal stakeholders, are prioritized by criticality into high, medium, or low priority recommendations. Within DAC, there is a strong focus on the high priority recommendations. Of the 1537 protocol review recommendations, 715 (47%) were considered high priority recommendations. High priority means that DAC believes the trial is at risk of ending uninformatively unless the issue is addressed before the study starts. The remaining 822 recommendations were considered medium (*n* = 240; 16%) and low (*n* = 582; 38%) priority. The median number of high priority recommendations per protocol review was 11.5 (range: 3 to 27), falling under a median of 6 (range: 3 to 9) distinct topics. Figure 3a shows the breakdown of all recommendations by priority within each topic. When examining only high priority recommendations (*n* = 715), 59% of recommendations fell into the top 3 topic areas, statistics and data analysis, trial procedures, and intervention and dose (Fig. 3b). Impact and trial design both had a relatively high proportion of high priority recommendations compared with medium and low priority, so when ordering by high priority recommendation counts rather than overall recommendation counts, these topics moved up to the 4^th^ and 5^th^ highest positions, respectively (Fig. 3b). The percentage increase in the proportion of recommendations represented by impact and trial design when moving from considering all recommendations to the subset of high priority recommendations was 46 and 74%, respectively. Of those grantees that have provided finalized trial protocols following DAC review (*n* = 32), 94% chose to implement at least one high priority review recommendation. Finalized trial protocols were compared with the protocol version originally submitted for DAC review to determine that 36% of all DAC recommendations for these protocols were implemented.

**Fig. 4:**
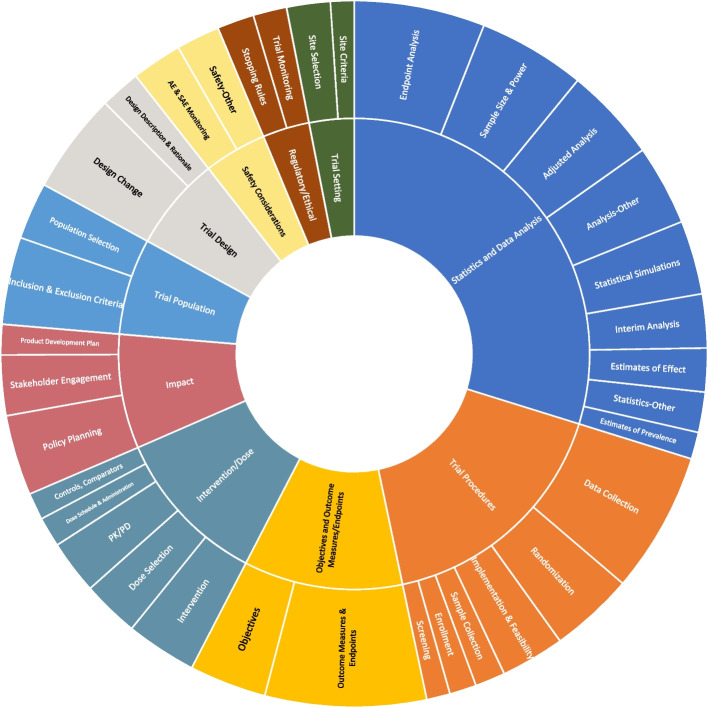
Protocol recommendation subtopics within topics for high priority recommendations comprising 1% or more of high priority recommendations (*n* = 651). Individual DAC recommendation statements were coded to subtopics shown in the outer circle based on the action recommended to be taken by the grantee. Specific subtopics were grouped into the high-level topics shown in the inner circle. The size of each segment represents the number of high priority recommendations that fell into that category. Only subtopics representing 1% or more of high priority recommendations are shown

Figure 4 is a visual representation of the high priority recommendation counts for the subtopics (shown in the outer ring) accounting for 1% or more of high priority recommendations in descending order within each topic category (shown in the inner ring). The size of each section corresponds to the number of recommendations within that subtopic or topic. Although it does not feature in one of the top 3 topic areas for high priority recommendation counts, the subtopic outcome measures and endpoints had the highest number of high priority recommendations overall, as indicated by it being the largest section in the outer ring. Within statistics and data analysis, the highest proportion of high priority recommendations were those relating to endpoint analysis (19%), sample size and power (16%), and adjusted analysis (14%). Within trial procedures, 73% of high priority recommendations were categorized as relating to data collection, randomization, and implementation and feasibility. Within intervention/dose, 63% of high priority recommendations were categorized as relating to intervention, dose selection, and PK/PD.

### Recommendations by trial phase

Of the 52 protocols submitted for review, 75% were for trials with an identifiable study phase according to the FDA definitions outlined on ClinicalTrials.gov [21]. The breakdown by study phase was as follows; phase 1: 15% (*n* = 8), phase 2: 19% (*n* = 10), phase 3: 19% (*n* = 10), phase 4: 21% (*n* = 11). For the remaining 13 studies (25%) where the study phase was not applicable, 10 were not interventional studies, and 3 were interventional studies where the phase did not apply because they were studies of devices or behavioral interventions. Out of the total number of review recommendations that were provided (*n* = 1537), 12% (*n* = 190) were for phase 1, 18% (*n* = 269) for phase 2, 18% (*n* = 277) for phase 3, 20% (*n* = 315) for phase 4, and 32% (*n* = 486) for those with no phase.

Some patterns appeared to emerge related to the trial phase and the focus of recommendations. For phase 1 trials, the most common recommendation fell under the subtopic PK/PD. This was the same when considering only the subset of high priority recommendations for phase 1 trials. For phase 2 trials, four subtopics had the highest number of recommendations overall (data collection, endpoint analysis, inclusion and exclusion criteria, and randomization). When considering the subset of high priority recommendations, dose selection was the most common subtopic for phase 2 trials. For phase 3 and phase 4 trials, endpoint analysis, and outcome measures and endpoints were the most common subtopics respectively. For high priority recommendations, the most common subtopics were outcome measures and endpoints for phase 3 trials, and statistical simulations for phase 4 trials. For studies with no phase, the most common subtopic for recommendations was adjusted analysis, whether looking at all recommendations or the subset of high priority recommendations.

**Fig. 5:**
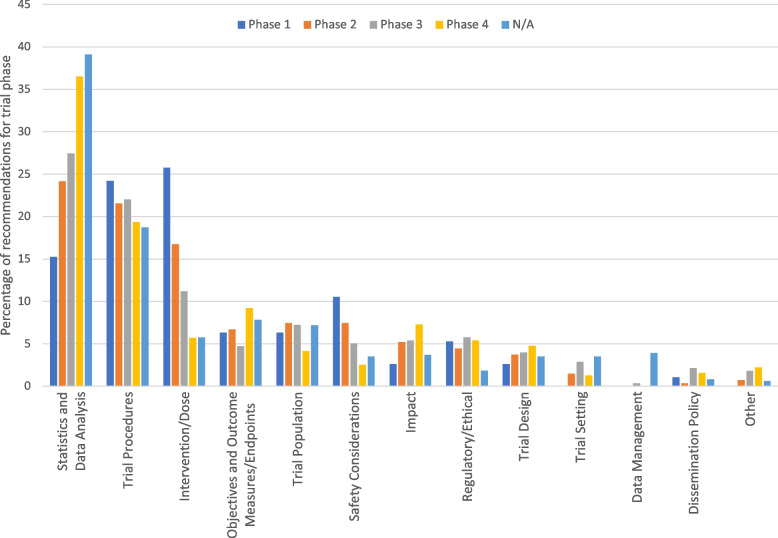
Percentage of recommendations for each topic within each clinical trial phase

Figure 5 shows the percentage of recommendations by topic within each clinical trial phase. Intervention/dose was the most common topic for recommendations for phase 1 trials, driven by the large number of recommendations pertaining to the subtopic, PK/PD. Recommendations relating to intervention/dose appeared to decrease in frequency with increasing trial phase. Similarly, recommendations related to safety considerations were at their highest within Phase 1 trials, dropping in frequency for phase, 2, 3, and 4 trials. This is consistent with the typical focus of phase 1 trials to establish a safety profile in healthy volunteers as well as the pharmacokinetic profile of the molecule. Conversely, there was a numerical increase in recommendations related to statistics and data analysis with the trial phase, and this was the most common recommendation topic for phase 2, 3, and 4 trials, as well as trials with no phase. Trial procedures appeared to account for a relatively high proportion of recommendations among all trial phases. For phase 1, 2, and 3 trials, the proportion of recommendations relating to trial procedures was only 1.6, 2.6, and 5.4%, lower than the most frequently recommended topic for each phase respectively. However, for phase 4 trials and trials with no phase, the relative proportion of statistics and data analysis compared with trial procedures (the next most common recommendation topic) appeared to increase more markedly (17.1% and 20.4%, respectively), due in part to increasing numbers of recommendations related to statistical simulations, sample size and power, adjusted analysis, and subgroup analysis. This seems reasonable given the potential for increasing statistical complexity and the increased propensity for potential confounders in phase 4 studies as well as observational studies which accounted for the majority of studies with no phase in our analysis.

## Discussion

This is the first study to analyze scientific review feedback provided to protocol authors by DAC, a BMGF program designed to enhance the informativeness of BMGF-funded clinical studies. Our frequency analysis showed that 1537 individual recommendations were made across 52 global health clinical trial protocols submitted to BMGF within a 3-year period. The majority of these protocols (90%) were for trials with at least one trial site situated in low- and middle-income countries (LMIC).

Adopters of a post-funding scientific design review process may have consistency in the types of trials for which they provide such reviews. This consistency could enable consolidation of the process across a few expert reviewers or best practice recommendations. In the case of this funder, the trials showed high dimensionality in many directions. The trials were interventional and non-interventional, for all phases of development, in the global South and North, across many disease areas, with complex and traditional designs. As a result, the breadth of expertise required to appropriately assess these protocols was extensive. Managing the scope of a new or growing program becomes critical when the necessary expertise spans a wide range of topics.

The recommendations covered a broad range of clinical trial design and informativeness areas (Fig. 4), highlighting the multidisciplinary expertise within DAC review teams. Done by others, a previous analysis of scientific review feedback on cancer trial protocol content found changes were requested for 33% of trials with a mean of 3.6 changes requested per trial, however for investigator-initiated trials, changes were more likely to be requested (54%) than for industry-sponsored studies (27%) [17]. The relatively high number of issues raised (median: 28 recommendations per trial) during protocol review in our analysis supports the assertion that a scientific review process may be particularly important for non-industry sponsored studies, and especially those in low resource settings such as the trials in our analysis, that may not have access to the same level of support and resources as in industry, or high resource settings.

In our analysis, the topic with the highest number of recommendations was statistics and data analysis, accounting for 31% of recommendations overall. Assuming the topic areas of the highest frequency are the likely areas where a protocol author team is weakest, this would imply those designing global health clinical trials as weak in biostatistics. Factors such as the high demand for such resources, their high costs, their low presence in LMIC, and the temptation to use traditional or simple techniques to obviate their need, all combine to increase the likelihood that these protocols are weak in this area. These dynamics, backed by anecdotal evidence, have led BMGF to augment clinical trial grants with planning grants specifically to fill biostatistical gaps in authoring teams.

The previous analysis of scientific review feedback on cancer trial protocol content conducted by others, categorized feedback into 5 topic areas (study design, intervention, population, evidence/rationale, or other) [17]. In that previous analysis, changes due to study design (“which included blinding, inclusion of placebo, randomization, stratification, selection of treatment arms, endpoints, assessments, monitoring, and statistical analysis plan”) were implemented in 40% of investigator-initiated trials after receiving scientific review feedback [17]. Most of the same issues included under the broad category study design in that analysis were incorporated as subtopics under either trial procedures or statistics and data analysis in our study, the two topics accounting for 52% of review recommendations. In our analysis, of those grantees who have provided an updated trial protocol, 94% chose to implement at least one of the high-priority DAC review recommendations in their updated trial protocol. Furthermore, in these updated trial protocols, 36% of all DAC recommendations were implemented. This level of implementation is one measure of potential impact, and the high proportion of trials with changes implemented demonstrates the utility of DAC reviews, since grantees are not required to adopt any DAC review recommendations. All trial protocols in our analysis received multiple recommendations, so we quantified individual recommendations, rather than the number of trials with changes requested. We chose a hierarchical categorization scheme to enable both a broad grouping of issues or topic areas (“topics”), as well as more granular detail (“subtopics”) to maximize the flexibility and potential utility of our data. This is also the first study, to our knowledge, to develop a detailed taxonomy defining distinct topic areas that can be used for classifying clinical trial review feedback.

The number and diversity of issues raised in our analysis lend further weight to previous recommendations calling for research sponsors to take an active role in ensuring their trials undergo a rigorous process of scientific review [5], particularly if those trials are not likely to receive such a review via other mechanisms [2]. Nearly half of all recommendations (47%) were characterized by the review team as high priority, meaning that DAC believes the study is at risk of ending uninformatively unless the issue is addressed before the study starts. Every protocol review included three or more high priority recommendations (median: 11.5; range: 3 to 27), falling under a median of 6 (range: 3 to 9) distinct topics per protocol review. This emphasizes the importance of a scientific review process at the post-funding stage of a trial when seeking to remedy the significant problem of uninformativeness in clinical research [2, 3]. Our data show that the review recommendations were not a fine polish or finalizing the details for these protocols. Almost two-thirds (65%) of protocols reviewed received at least one recommendation on the topic of trial design (Table 5). This topic is unique in that it is often the first attribute described about a trial and the hub from which many other decisions flow. As the chassis of the trial, if recommendations are made to change the design, a cascade of other decisions comes into question. A need to change the trial design implies some real opportunity was left on the table when the original design was conceived.

In making their recommendations, DAC reviewers are guided to consult a selection of trial “best practices” for informativeness (Table 2). At times, this means that recommendations not only relate directly to the individual trial design, but they also relate to many other relevant contextual factors that will have an impact on whether the trial ultimately contributes in a meaningful way within a global policy and research context. In our analysis, many of these recommendations were captured under three distinct subtopics: policy planning, stakeholder engagement, and product development plan. Overall, 75 recommendations across the 52 protocols related to any one of these three subtopics were incorporated into a topic called “Impact”. These types of recommendations encouraged protocol authors to explicitly consider the next steps in the development of the intervention under investigation, and the potential impact of their study outcomes on policy. This included advising protocol authors to consult with relevant stakeholders early in the trial design process to ascertain the pathway to policy or practice change that would be supported by their study. The inclusion of specific recommendations to guide authors toward maximizing the impact of their research, alongside recommendations targeted toward study design and analysis issues indicates alignment with previously published criteria to enhance the informativeness of clinical trials [2].

One limitation of our analysis is the reliance on quantifying the number of individual recommendations relating to topic areas. Some areas are more conceptually complex than others and are therefore more likely to have a higher number of individual recommendations. For example, statistics and data analysis arguably comprises many more technical concepts than an area such as trial population or trial setting. This affects the possibilities for distinct recommendations. Further, some topic areas may lend themselves to more interpretive variance. An area with high conceptual complexity and high interpretive variance from expert to expert will likely lead to a much greater recommendation volume. While this may inflate the number of recommendations for certain topics, we believe the conceptual complexity that leads to a higher recommendation count is also an accurate reflection of the need for expertise in that area when designing a clinical trial or performing a scientific review of a trial protocol. This limitation is one reason that we chose to classify recommendations according to 58 specific subtopics. A detailed taxonomy with definitions used for classification is provided to enhance transparency and reproducibility. This allows readers to see the composition of the broad topics and use the more granular subtopics for interpretation.

Recommendation data was one source used to generate subtopics for this analysis. This could be considered a limitation as it would be possible for important areas of focus to be missing if they were not raised by the DAC review teams. However, this approach was supplemented with consultation of protocol development guidance and templates to ensure that the main topics and subtopics were captured. These guidance documents were also used to decide where subtopics should be placed within the high-level topics. This iterative process to determine and define subtopics using a combination of raw data as well as known protocol development standards enabled the development of a more comprehensive taxonomy of subtopics into a codebook that could be used for manual text classification of our dataset. The transparency in providing clear and delineated subtopic definitions means that the process is reproducible, increasing the potential utility of our codebook for others, and allows for appropriately documented changes to the subtopics in future analyses. An organization considering setting up a scientific review of a clinical trial might be well-served to use our codebook, either to guide new reviewers toward a priori focus areas, or to typify recommendations post-review.

A further limitation is based the need to honor the sensitivities of protecting BMGF relationships with grantees and their information. A variety of potentially interesting additional information could not be included here as a result. Information such as grantee institutions, names of specific studies, excerpts from submitted protocols, excerpts from reviews, or reviewer comments directed confidentially to specific grantees would or could represent identifiable information or be a breach of confidentiality. In addition, information on some BMGF or DAC practices, organization, and approaches would be considered proprietary. Information restrictions in these cases preclude the inclusion of certain data on how DAC engages reviewers and partners.

This analysis reports descriptive statistics generated from a process of manual text classification. As DAC began expert protocol reviews relatively recently in 2020, this analysis was an opportunity to audit DAC protocol review feedback over its first 3 years of operation and consider the characteristics and frequency distribution of review recommendations made to protocol authors. Very few publications have described actual feedback provided to protocol authors resulting from a post-funding scientific review process. The purpose of our analysis was primarily to describe the nature and scope of DAC protocol review recommendations. The feedback themes identified in our analysis can offer insight into the specific areas that should be considered when reviewing clinical trial protocols from a scientific perspective. For example, when forming scientific review panels, consideration should be given to the clinical phase of trial protocols under review. Ideally, the composition of a review panel will reflect the expertise pertinent to different stages in the development of an intervention.

Our analysis also highlights potential areas of trial design weakness in global health clinical trials, which may be instructive when considering the support best offered in the early stages of such trials to increase the likelihood of an informative outcome. This dataset may continue to be augmented and used for future analyses to explore questions about the types of design changes that are being recommended, at what frequency, and to notice trends in recommendations over time.

## Conclusions

This is the first analysis of DAC scientific review feedback provided to protocol authors for BMGF-funded studies. This study provides a detailed taxonomy of scientific review feedback topic areas that can be used to categorize clinical trial design topics. The hierarchical approach provides additional granularity and flexibility for using the data. The areas with the highest frequency of recommendations were statistics and data analysis, trial procedures, and intervention/dose; however, there was some deviation when the data was examined by trial phase. A focus on enhancing trial informativeness meant that recommendations related not only to design and analysis issues in the trial protocol, but also to the likely policy impact of the study. The high number of recommendations made per protocol review across a number of distinct topic areas highlights the potential value of a distinct scientific review to enhance the informativeness of global health clinical trials. To maximize utility this scientific review must take place prior to trial initiation, and review teams should comprise statistical and trial design expertise with additional expertise tailored to the trial/intervention type and phase. Research progress to new solutions builds from multiple independent studies. Study quality in any single study can be critical to all. All funders ought to add forensic, protocol-level scientific design reviews to their existing offerings.

## Supplementary Information

Additional file 1. Additional methods detail on codebook development. Additional detail describing the process of inductive and deductive content analysis to develop the codebook, as well as the iterative process of refining the codebook following initial manual text classification.

Additional file 2. Codebook for manual text classification of subtopics. Codebook used for assigning subtopics to recommendation statements, according to the definitions shown. The topic column shows the parent category for each subtopic in the hierarchical coding exercise.

Additional file 3. Protocol review recommendation counts by subtopic in descending order. Table showing complete counts for the number of recommendations falling under each subtopic.

## Data Availability

The data analyzed during the current study is internal and confidential to the BMGF and as such is not available.

## Abbreviations

BMGF: Bill and Melinda Gates Foundation
DAC: Design, Analyze, Communicate group
FDA: U.S. Food and Drug Administration
ICH: International Council for Harmonisation of Technical Requirements for Pharmaceuticals for Human Use
IRB: Institutional Review Board
LMIC: Low- and middle-income countries
NHS: National Health Service
NIH: National Institutes of Health
OL: Opportunities List
PK/PD: Pharmacokinetics/pharmacodynamics
PR: Protocol review
SME: Subject Matter Expert
WHO: World Health Organization

## References

[1] Hartman D, Heaton P, Cammack N, Hudson I, Dolley S, Netsi E, et al. Clinical trials in the pandemic age: what is fit for purpose? Gates Open Res. 2020;9(4):58. 10.12688/gatesopenres.13146.1 PMC7324688 32656501

[2] Zarin DA, Goodman SN, Kimmelman J. Harms from uninformative clinical trials. JAMA. 2019;322(9):813. 31343666 10.1001/jama.2019.9892

[3] Hutchinson N, Moyer H, Zarin DA, Kimmelman J. The proportion of randomized controlled trials that inform clinical practice. eLife. 2022;17(11):e79491. 10.7554/eLife.79491 PMC9427100 35975784

[4] Yordanov Y, Dechartres A, Porcher R, Boutron I, Altman DG, Ravaud P. Avoidable waste of research related to inadequate methods in clinical trials. BMJ. 2015;350(mar24 20):h809–h809. 25804210 10.1136/bmj.h809 PMC4372296

[5] Dolley S, Norman T, McNair D, Hartman D. A maturity model for the scientific review of clinical trial designs and their informativeness. Public Health and Healthcare; 2023. Available from: https://www.preprints.org/manuscript/202304.0147/v1. Cited 2023 Oct 14. 10.1186/s13063-024-08099-5 PMC11027356 38641848

[6] Bendiscioli S. The troubles with peer review for allocating research funding: funders need to experiment with versions of peer review and decision-making. EMBO Rep. 2019;20(12):e49472. 31680417 10.15252/embr.201949472 PMC6893288

[7] Hug SE, Aeschbach M. Criteria for assessing grant applications: a systematic review. Palgrave Commun. 2020;6(1):37.

[8] Recio-Saucedo A, Crane K, Meadmore K, Fackrell K, Church H, Fraser S, et al. What works for peer review and decision-making in research funding: a realist synthesis. Res Integr Peer Rev. 2022;7(1):2. 35246264 10.1186/s41073-022-00120-2 PMC8894828

[9] Turner S, Bull A, Chinnery F, Hinks J, Mcardle N, Moran R, et al. Evaluation of stakeholder views on peer review of NIHR applications for funding: a qualitative study. BMJ Open. 2018;8(12):e022548. 30552252 10.1136/bmjopen-2018-022548 PMC6303555

[10] McNair L. Ethical and regulatory oversight of clinical research: the role of the institutional review board. Exp Biol Med (Maywood). 2022;247(7):561–6. 35172623 10.1177/15353702221078216 PMC9014527

[11] Binik A, Hey SP. A framework for assessing scientific merit in ethical review of clinical research. Ethics Hum Res. 2019;41(2):2–13. 30895755 10.1002/eahr.500007

[12] NIH Office of Clinical Research. Available from: https://ocr.od.nih.gov/pdfs/2019_Revised_Policy_Scientific_Review_Protocols_FINAL_revised06192019.pdf.

[13] Ning N, Yan J, Dietrich MF, Xie XJ, Gerber DE. Institutional scientific review of cancer clinical research protocols: a unique requirement that affects activation timelines. JOP. 2017;13(12):e982–91. 29019706 10.1200/JOP.2017.024299 PMC5728362

[14] Lardot C, Steward W, Van Glabbeke M, Armand JP. Scientific review of EORTC trials. Eur J Cancer. 2002;38:24–30. 10.1016/s0959-8049(01)00453-1 11858960

[15] Bill & Melinda Gates Foundation. 2023. Available from: https://www.gatesfoundation.org/about.

[16] DAC assessment tool. Available from: https://dac-trials.tghn.org/resources/dac-assessment-tool/dat-doi-landing-page/.

[17] Ning N, Yan J, Xie XJ, Gerber DE. Impact of NCI-mandated scientific review on protocol development and content. J Natl Compr Canc Netw. 2015;13(4):409–16. 25870377 10.6004/jnccn.2015.0056 PMC5119526

[18] ICH. ICH clinical electronic structured harmonised protocol M11 template. 2022. Available from: https://www.ema.europa.eu/en/documents/scientific-guideline/ich-m11-template-step-2b_en.pdf.

[19] Council for International Organizations of Medical Sciences (CIOMS) in collaboration with the, World Health Organization (WHO). International ethical guidelines for health-related research involving humans. Available from: https://cioms.ch/wp-content/uploads/2017/01/WEB-CIOMS-EthicalGuidelines.pdf. 40523065

[20] WHO. Recommended format for a “research protocol”. Available from: https://www.who.int/groups/research-ethics-review-committee/recommended-format-for-a-research-protocol.

[21] ClinicalTrials.gov. Protocol registration data element definitions for interventional and observational studies. Available from: https://prsinfo.clinicaltrials.gov/definitions.html.

[22] NHS Health Research Authority. Protocol guidance and template tool. Available from: https://www.hra.nhs.uk/planning-and-improving-research/research-planning/protocol/.

[23] World Bank. New World Bank country classifications by income level: 2022-2023. Available from: https://blogs.worldbank.org/opendata/new-world-bank-country-classifications-income-level-2022-2023. Cited 2023 Oct 13.

[24] Landis JR, Koch GG. The measurement of observer agreement for categorical data. Biometrics. 1977;33(1):159–74. 843571

[25] Fleiss JL. Statistical methods for rates and proportions. 1st ed. London: John Wiley & Sons; 1981. p. 218.

