# Supplementary material for: Scientific review of protocols to enhance informativeness of global health clinical trials
Source: Trials. 2025 Mar 12;26:85. doi: 10.1186/s13063-025-08763-4 (PMC11899556; doi:10.1186/s13063-025-08763-4)
Supplement: Supplementary file 1 — Additional file 1. Additional methods detail on codebook development. Additional detail describing the process of inductive and deductive content analysis to develop the codebook, as well as the iterative process of refining the codebook following initial manual text classification. [file 13063_2025_8763_MOESM1_ESM.pdf]

Additional File 1

Additional methods detail on codebook development

The highest-level headings from both the ICH and NHS clinical trial protocol templates were compared, and topics that were the same in meaning were aligned across both documents. Headings that were only relevant in the context of the protocol document, such as "Introduction", "References", and "Appendices" were ignored, as they do not represent meaningful topics for protocol feedback which is typically not focused on the presentation or structure of the protocol document. Subtopics generated from the initial analysis of source data were mapped to the ICH and NHS protocol template headings where possible. Protocol templates include a structured hierarchy of subheadings and specific information about the contents of each section of a protocol. The documented guidance for each protocol template and the subheading structures from ICH and NHS were used to determine how a particular DAC subtopic would be categorized to a topic. Headings that were present in one document (either NHS or ICH) but not the other were reviewed against the list of subtopics generated from the source data to determine whether that heading should be included as a high-level topic. Subtopics derived from the source data that did not appear to fit in either the ICH or NHS protocol template headings fit one of two cases. At times, they were placed within one of the existing high-level topics (for example, subtopics "Implementation and feasibility" and "Community engagement" were placed under the topic "Trial procedures"). Alternately, —where they were significantly different from the headings— they were used to generate additional high-level topics ("Impact" and "Other"). Where there were different options for the placement of a subtopic between ICH and NHS guidance, knowledge of what would generally meet the expectations of most expert scientific review teams

who had provided the source data feedback was used to make a final decision. All identified subtopics were assembled in a codebook and definitions were added to each subtopic. The definition for each subtopic states the concepts and types of recommendations that would fall under that subtopic.

Decisions for the level of specificity in subtopics were based on a few different factors: the types of subheadings that were present in protocol development templates; the likely proportion of recommendations that would fall under a given subtopic; as well as whether it would be feasible to delineate the recommendation statements into different subtopics. This depended somewhat on the conceptual complexity of each subtopic, and whether that complexity was likely to be expressed in an independent way among different recommendation statements to allow for clear delineation. The source data analysis approach to subtopic identification combined with the structured, exogenous topics meant that some topics included very few subtopics (for example, "Trial setting") while other topics included many subtopics (for example, "Statistics and data analysis").

Codebook development was an iterative process. Following the completion of the manual text classification process described in the methods section of the main publication, it was observed that one topic ("Intervention/dose") with few subtopics, accounted for 10% of the total number of recommendations (the 3rd highest proportion of recommendations for a topic). As this topic only had three subtopics ("Intervention", "Dosing", "PK/PD"), it was decided that a greater level of detail would benefit the analysis of these recommendations. ICH protocol subheadings within the section called "Trial Intervention and Concomitant Therapy" were used to inform the

generation of additional more specific subtopics related to intervention and dose. Furthermore, where topics had only a single subtopic and accounted for 2% or more of recommendations ("Objectives and outcome measures/endpoints"; "Trial design"; "Trial setting"; "Trial population"; "Safety considerations"), these topics were subdivided into more specific subtopics to provide greater clarity on the types of recommendations made within these topics. For example, the topic "Trial design" was subdivided into three subtopics: "Design change", "Design description and rationale", and "Design timepoints". The selection of additional subtopics was informed by the subheadings within ICH protocol development guidance that were relevant to the source data recommendations.

For cases where a recommendation was identified that did not seem to fit within any of the subtopic definitions, a best choice was made from among the existing subtopics, however a note was added suggesting a proposed new subtopic. After initial coding was completed for all recommendations, both coders reviewed the cases where it seemed that the source data warranted inclusion of a new subtopic to enhance the comprehensiveness of the codebook. A consensus approach was used to identify a small number of new subtopics which were subsequently defined, added to the codebook, and consistently applied to the dataset. The iterative nature of codebook development allowed for quality enhancement and ensured that subtopic identification was driven by the entirety of the source data.
