# Supplementary material for: Scientific review of protocols to enhance informativeness of global health clinical trials
Source: Trials. 2025 Mar 12;26:85. doi: 10.1186/s13063-025-08763-4 (PMC11899556; doi:10.1186/s13063-025-08763-4)
Supplement: Supplementary file 2 — Additional file 2. Codebook for manual text classification of subtopics. Codebook used for assigning subtopics to recommendation statements, according to the definitions shown. The topic column shows the parent category for each subtopic in the hierarchical coding exercise. [file 13063_2025_8763_MOESM2_ESM.pdf]

Additional File 2 Codebook used for assigning subtopics to recommendation statements, according to the definitions shown. The topic column shows the parent category for each subtopic in the hierarchical coding exercise.

Topic	Subtopic	Definition
Objectives and outcome measures/endpoints	Objectives	Recommendations related to the study objectives, including recommendations to change the study objectives, define them more clearly, or to provide a rationale for the choice of study objectives.
Objectives and outcome measures/endpoints	Outcome measures and endpoints	Recommendations related to the choice of outcomes and endpoints to meet the study objectives, including the ways to measure them.
Trial design	Design change	Recommendations requesting modifications or changes to the study design. Does not include recommendations related to the trial duration or other key timepoints in the trial which fall under 'Design Timepoints'. Does not include recommendations asking for more detail or to provide a rationale for trial design decisions (these fall under 'Design Description and Rationale').
Trial design	Design description and rationale	Recommendations asking for a more detailed description of the trial design, or to provide a rationale for trial design decisions. Does not include decisions related to duration or trial timing which would fall under 'Key Timepoints'. Does not include recommendations for changes to the trial design which fall under 'Design Change'.
Trial design	Design timepoints	Recommendations related to the rationale for trial timing or duration, or other key timepoints in the trial where they are a significant part of the trial design.
Trial setting	Site criteria	Recommendations about the characteristics or facilities that sites should have to enable participation in the study, without recommending particular sites or recommending that sites be added or removed. Suggestions or requests to provide additional information on the attributes of all or specific sites are also included here. More specific recommendations for the inclusion or exclusion of particular sites, or general recommendations for sites to be added or removed fall under 'Site Selection'.
Trial setting	Site selection	Recommendations related to whether or not specific sites or areas should be part of the proposed study, including recommendations to add or remove sites from the study. Note that specific recommendations related to estimates of disease burden (prevalence/incidence) are under 'Estimates of Prevalence'.
Trial population	Inclusion and exclusion criteria	Recommendations related to the specific inclusion/exclusion criteria for the trial population. This may include asking for clarification on certain criteria, or recommending certain groups be included or excluded for various reasons.
Trial population	Population selection	Recommendations related to the selection of the trial population, including the rationale for the selected population.
Intervention/dose	Concomitant therapies	Recommendations related to any concomitant therapies, whether they are allowed, asking to ensure they are not used, or mitigate the situation where they are used.
Intervention/dose	Controls, comparators	Recommendations about the choice of control/comparator. Includes recommendations for using particular comparators, as well as recommendations asking for rationale for choice of comparator.
Intervention/dose	Dose schedule and administration	Recommendations about the timing of the dosing regimen, dosing instructions or how doses should be administered.

Topic	Subtopic	Definition
Intervention/dose	Dose selection	Recommendations about specifically what dose regimens will be given in any of the study arms - asking for clarification on what is the amount/concentration, or recommending particular amounts/concentrations or changes to doses. Also includes recommendations related justifying the rationale for the choice of dose for any arm. This is distinct from comments about the design of a dose-finding study which would fall under 'Trial Design'.
Intervention/dose	Dose-other	Recommendations related to dose that fall outside of other more specific dose categories ('Dose Schedule and Administration', 'Dose Selection', 'PK/PD', 'Toxicity').
Intervention/dose	Intervention	Recommendations related to the choice of intervention. Includes recommendations asking for more detail on what it is, or to modify aspects of the intervention, or to justify choice of intervention.
Intervention/dose	Intervention compliance	Recommendations about assessing/ensuring compliance with the intervention.
Intervention/dose	Intervention-other	Recommendations related to intervention that fall outside of other more specific intervention categories ('Concomitant Therapies', 'Controls, Comparators', 'Intervention', 'Intervention Compliance', 'Prep, Handling, Storage').
Intervention/dose	PK/PD	Recommendations for PK/PD analyses and/or PK/PD considerations. If the recommendation relates to a particular objective/endpoint of the study that happens to be a PK objective/endpoint, this would fall under 'Objectives' or 'Outcome Measures and Endpoints'.
Intervention/dose	Prep, handling, storage	Recommendations related to the preparation, handling, storage of intervention components, including where products are sourced from, as well as ensuring the quality control of products.
Intervention/dose	Toxicity	Recommendations about dose specifically related to toxicity, or recommendations mentioning how to choose a dose that minimizes toxicity.
Trial procedures	Baseline assessments	Recommendations related to any assessments or procedures to be conducted at baseline.
Trial procedures	Community engagement	Recommendations related to communication with study participants or members of the community to increase engagement with the study.
Trial procedures	Data collection	Data refers to information or measurements collected from participants that are not a biological sample (see 'Sample Collection'). This category includes recommendations that particular types of data or information are collected from participants at different timepoints during the study, except at baseline (these fall under 'Baseline Assessments'). Also includes comments related to the rationale for the choice of data collection or the schedule of data collection. Recommendations regarding the way that data are collected are also included. This is distinct from choice of measurement as this is about the mechanism for collecting relevant participant data rather than the way an endpoint is measured.
Trial procedures	Enrollment	Recommendations related to the way participants are identified and recruited (including any intended incentives for participants).
Trial procedures	Implementation and feasibility	Recommendations related to implementation/feasibility considerations for the trial/intervention where an action does not fit under another topic.

Topic	Subtopic	Definition
Trial procedures	Long term follow-up	Recommendations related to planning for long term follow-up after the study.
Trial procedures	Randomization	Recommendations regarding the randomization process. This includes generating the randomization sequence, as well as allocation, blinding, and any other steps related to the implementation of randomization. This includes comments about who will be blinded after assignment to interventions and how that will be maintained. Also includes comments about stratification, as well as cluster definitions.
Trial procedures	Retention	Recommendations related to retention of participants in the trial, including strategies for increasing retention.
Trial procedures	Sample collection	Recommendations related to biological samples that are to be taken from participants at different timepoints during the study, except at baseline (these fall under 'Baseline Assessments'). Also includes comments related to the rationale for choice of sample collection or the schedule of sample collection. Recommendations regarding the way that samples are collected are also included. This is distinct from choice of measurement as this is about the mechanism for collecting the relevant samples rather than the way an endpoint is measured.
Trial procedures	Screening	Recommendations related to the screening of participants to determine eligibility for study entry, including any screening procedures and how they are implemented effectively.
Trial procedures	Withdrawal criteria	Recommendations related to the circumstances and process for how participants will be withdrawn from the trial, whether participants are to be replaced, and any follow up of withdrawn participants.
Statistics and data analysis	Adjusted analysis	Recommendations about adjustments to be made in analyses. This includes comments about covariates to be included in analyses and models.
Statistics and data analysis	Analysis-other	Recommendations related to analysis that fall outside of other more specific analysis categories (Endpoint, Adjusted, Subgroup, Interim).
Statistics and data analysis	Endpoint analysis	Recommendations for how to analyze endpoints.
Statistics and data analysis	Estimates of effect	Recommendations related to estimates of the expected effect size prior to conducting the trial.
Statistics and data analysis	Estimates of prevalence	Recommendations related to estimates of prevalence, incidence of disease, and disease burden in the areas chosen for the study.
Statistics and data analysis	Interim analysis	Recommendations related to conducting an interim analysis, including for the purpose of sample size re-assessment, or stopping the trial for futility, success or safety reasons.
Statistics and data analysis	Missing data	Recommendations related to the way that missing data will be handled.
Statistics and data analysis	Sample size and power	Recommendations related to sample size calculations and rationale, as well as recommendations related to assumptions for power calculations for the study to be successful.
Statistics and data analysis	SAP	Recommendations asking investigators to make sure a SAP is completed prior to the study.
Statistics and data analysis	Statistical simulations	Comments specifically recommending the use of simulations to optimize various aspects of the study design.
Statistics and data analysis	Statistics-other	Recommendations related to statistical methods that fall outside of other statistical categories (Endpoint Analysis, Adjusted Analysis, Subgroup Analysis, Interim Analysis, Analysis-Other, SAP, Missing Data).
Statistics and data analysis	Subgroup analysis	Recommendations about subgroup analyses, including which subgroups to conduct separate analyses in, and how to do it.

Topic	Subtopic	Definition
Safety considerations	AE and SAE monitoring	Recommendations related to adverse event monitoring, including defining adverse events and serious adverse events, as well as recommendations for how and when to collect adverse event information.
Safety considerations	Safety assessments	Recommendations related to safety assessments that would not fall under AE and SAE Monitoring. For example, physical examinations, vital signs, and other assessments that are specifically related to safety, but are not strictly adverse event monitoring.
Safety considerations	Safety-other	Recommendations related to safety considerations that do not fall under 'Safety Assessments' or 'AE and SAE Monitoring', and that would also not fall under a topic related to pausing or stopping the trial for safety, such as 'Interim Analysis', 'Stopping Rules' or 'Trial Monitoring'.
Regulatory/ethical	Consent	Recommendations related to the procedure of obtaining informed consent from participants, or the inclusion of certain information as part of the consent process.
Regulatory/ethical	Ethical considerations	Recommendations related specifically to ethical considerations for the trial, that do not fall under another subtopic, for example addressing potential unintended consequences of trial participation for participants.
Regulatory/ethical	Stopping rules	Recommendations related to trial stopping rules where they do not fit under 'Interim Analysis'.
Regulatory/ethical	Trial monitoring	Recommendations relating to any kind of trial monitoring (e.g., by a data monitoring committee), where the recommendation would not fall under 'Interim Analysis' or 'Stopping Rules'.
Impact	Policy planning	Recommendations about planning for a pathway to policy change. Recommendations related to ensuring that the study has the desired impact on policy, where those recommendations do not fall under Stakeholder Engagement, or other basic actions that may lead to policy change.
Impact	Product development plan	Recommendations related to ensuring the current trial fits into the overall development plan for the product or intervention. Also includes sustainability considerations.
Impact	Stakeholder engagement	Recommendations advising consultation with regulatory bodies or policy/guideline development bodies to increase likelihood that the study provides necessary data to change policy/guidelines.
Data management	Data management	Recommendations related to data quality assurance and management of trial data.
Dissemination policy	Dissemination	Recommendations related to the dissemination of trial data or publications that are not related specifically to open access availability.
Dissemination policy	Open access	Recommendations specifically related to making the trial data or publications available in an open access resource.
Other	Other	Recommendations that do not fall under any other categories.
Other	Other bias	Comments raising potential bias issues (for example, confounding, contamination) without a recommendation or preference stated for how to mitigate it.
