# Supplementary material for: Scientific review of protocols to enhance informativeness of global health clinical trials
Source: Trials. 2025 Mar 12;26:85. doi: 10.1186/s13063-025-08763-4 (PMC11899556; doi:10.1186/s13063-025-08763-4)
Supplement: Supplementary file 3 — Additional file 3. Protocol review recommendation counts by subtopic in descending order. Table showing complete counts for the number of recommendations falling under each subtopic. [file 13063_2025_8763_MOESM3_ESM.pdf]

Additional File 3 Protocol review recommendation counts by subtopic in descending order.

**Topic**	**Subtopic**	**Total**
Trial procedures	Data collection	100
Objectives and outcome measures/endpoints	Outcome measures and endpoints	82
Statistics and data analysis	Endpoint analysis	79
Statistics and data analysis	Adjusted analysis	68
Statistics and data analysis	Analysis-other	62
Statistics and data analysis	Sample size and power	58
Trial population	Inclusion and exclusion criteria	56
Trial procedures	Implementation and feasibility	51
Trial procedures	Randomization	51
Intervention/dose	Intervention	48
Trial population	Population selection	44
Trial design	Design change	38
Intervention/dose	PK/PD	38
Trial procedures	Sample collection	37
Safety considerations	AE and SAE monitoring	36
Statistics and data analysis	Statistical simulations	35
Statistics and data analysis	Missing data	34
Impact	Stakeholder engagement	30
Impact	Policy planning	30
Objectives and outcome measures/endpoints	Objectives	28
Statistics and data analysis	Statistics-other	26
Statistics and data analysis	Subgroup analysis	26
Statistics and data analysis	Interim analysis	25
Trial procedures	Enrollment	24
Safety considerations	Safety-other	24
Statistics and data analysis	SAP	23
Intervention/dose	Dose selection	22
Statistics and data analysis	Estimates of effect	21
Data management	Data management	20
Regulatory/ethical	Stopping rules	20
Trial setting	Site selection	20
Regulatory/ethical	Trial monitoring	20
Regulatory/ethical	Consent	19
Safety considerations	Safety assessments	19
Statistics and data analysis	Estimates of prevalence	18
Trial procedures	Screening	17
Intervention/dose	Dose schedule and administration	16
Trial procedures	Community engagement	15
Impact	Product development plan	15
Dissemination policy	Open access	14
Trial design	Design description and rationale	13
Intervention/dose	Controls, comparators	13
Trial setting	Site criteria	13
Intervention/dose	Intervention compliance	11
Other	Other bias	9
Trial procedures	Retention	9
Other	Other	8
Intervention/dose	Prep, handling, storage	8
Trial procedures	Baseline assessments	7
Trial design	Design timepoints	7
Intervention/dose	Concomitant therapies	6
Regulatory/ethical	Ethical considerations	5
Intervention/dose	Toxicity	4
Dissemination policy	Dissemination	4
Intervention/dose	Intervention-other	4
Trial procedures	Withdrawal criteria	3
Trial procedures	Long term follow-up	3
Intervention/dose	Dose-other	1
	**Total**	**1537**
